# Cognitive function in a general population of men and women: a cross sectional study in the European Investigation of Cancer–Norfolk cohort (EPIC-Norfolk)

**DOI:** 10.1186/1471-2318-14-142

**Published:** 2014-12-19

**Authors:** Shabina A Hayat, Robert Luben, Stephanie Moore, Nichola Dalzell, Amit Bhaniani, Serena Anuj, Fiona E Matthews, Nick Wareham, Kay-Tee Khaw, Carol Brayne

**Affiliations:** Department of Public Health and Primary Care, Institute of Public Health, University of Cambridge School of Clinical Medicine, Cambridge, UK; MRC Biostatistics Unit, Institute of Public Health, Cambridge, UK; MRC Epidemiology Unit, University of Cambridge School of Clinical Medicine, Cambridge Biomedical Campus, Cambridge, CB2 0QQ UK

**Keywords:** Ag(e)ing, Follow up studies, United Kingdom, Middle aged, Cognition, Cognition disorders

## Abstract

**Background:**

Although ageing is strongly associated with cognitive decline, a wide range of cognitive ability is observed in older populations with varying rates of change across different cognitive domains.

**Methods:**

Cognitive function was measured as part of the third health examination of the European Prospective Investigation of Cancer in Norfolk (EPIC-Norfolk 3) between 2006 and 2011 (including measures from the pilot phase from 2004 to 2006). This was done using a battery consisting of seven previously validated cognitive function tests assessing both global function and specific domains. The battery included a shortened version of the Extended Mental State Exam (SF-EMSE); letter cancellation task; Hopkins Verbal Learning Test (HVLT); Cambridge Neuropsychological Test Automated Battery Paired Associates Learning Test (CANTAB-PAL); Visual Sensitivity Test (VST); Shortened version of the National Adult Reading Test (Short-NART) and a task to test for prospective memory. We report the distribution of cognitive function in different cognitive domains by age and sex and compare the utility of a number of assessment tests in a general population of older men and women.

**Results:**

Cognitive test data were available for 8585 men and women taking part in EPIC-Norfolk 3. Increasing age was generally associated with declining mean cognitive function, but there was a wide range observed within each age group as well as variability across different cognitive domains. Some sex differences were also observed.

**Conclusion:**

Descriptive data are presented for this general population sample of older men and women. There is a wide range of cognitive performance seen in this population. Though average performance declines with age, there is large individual variability across different cognitive domains. These variations may provide insights into the determinants of cognitive function in later life.

**Electronic supplementary material:**

The online version of this article (doi:10.1186/1471-2318-14-142) contains supplementary material, which is available to authorized users.

## Background

Ageing is generally associated with memory impairment and cognitive decline, however, this decline is not inevitable [1] and not all domains of cognitive function are equally affected with age [2]. A broad range of cognitive capability is observed in the older population [3] as well as substantial inter-individual heterogeneity in rates of decline [4]. The range encompasses high cognitive functioning even in the very old [5], mild cognitive impairment (MCI), through to dementia at the other end of the spectrum.

Mild Cognitive Impairment (MCI) is described as the transitional state between normal cognitive ageing and dementia [6], with detectable impairment to memory or cognitive abilities when compared to healthy controls, but not to the extent as seen in individuals suffering with mild dementia [7]. The amnestic form of MCI has been shown to be predictive of dementia [7–10], however, the conversion rate has been shown to vary significantly [7] and only a minority of individuals with MCI progress to dementia within clinically relevant time frames. As a result of the heterogeneity in both the aetiology and outcome of this condition, MCI is still an area of major debate, with no consensus on its classification [11]. Reliable identification of those individuals with MCI who remain stable compared to those who decline would maximise efficacy of potential treatments and preventive interventions around this transitional stage [12]. To determine the factors that contribute to this variability in not just the rates of decline in MCI, but also in the different cognitive abilities, will help to improve the understanding of the natural progression of decline in an ageing population.

Substantial data already exist on dementia and cognitive impairment, mainly in the older population, from using a wide range of instruments, each with merits and limitations that assess different aspects or stages of cognition. Episodic memory deficits have been shown in a number of studies to be associated with the strongest and most persistent risk of cognitive decline [13, 14] and are the most common and earliest complaints in MCI [15]. However deficits in other cognitive domains can also occur, some early on, including attention, executive functioning, prospective memory, semantic memory, verbal ability, visuospatial skills, attention and processing speed [16–19]. There is a need for assessments to cover a broad range of ability and domains, and have an optimal balance between sensitivity and specificity with high positive predictive value in the settings in which they will be applied.

There are no cures for dementia, but there are some advances in the development of drugs [20] that are known to improve symptoms, or temporarily slow down disease progression in early and middle stages of the disease. To exploit the potential benefits of any such treatment and to facilitate decision making for future plans, it is important to identify early indicators of decline. However, before we are able to advise guidelines and policies on health, we need to gain better insight into the ageing process in the general population and the range of functions in both domain specific and global cognition.

The primary aim of this study was to obtain data from a general population of men and women without overt cognitive impairment using a comprehensive cognitive test battery assessing a range of function including memory (retrospective and prospective), executive function, attention, calculation, registration, language, praxis, abstract thinking, processing and new learning. The secondary aim was to explore the comparability of the different tests and their use in a community setting. We focused on producing a standardised protocol for test administration and scoring, thus minimising variation, differences in interpretation and reducing subjectivity. Details of the standardisation methods are given here.

## Methods

The European Prospective Investigation of Cancer (EPIC) is a 10-country collaboration studying diet and disease with half a million participants [21, 22], of which EPIC-Norfolk is one of the UK centres. Detailed descriptions of recruitment and study methods at baseline have been reported elsewhere [23]. Briefly, participants were recruited at baseline through registers in thirty five general practices in Norfolk between 1993 and 1997. General practice registers approximate population based registers as virtually all the population are registered through the UK National Health Service. Participants who consented at baseline were re-invited for a health examination at subsequent phases. The data presented here is from the third health examination (3HC or EPIC-Norfolk 3) conducted between 2006 and 2011 (including data from the pilot phase between 2004 and 2006) [24]. The characteristics of the participants taking part in EPIC-Norfolk 3 are given in Table 1. Attrition rates and characteristics of these returning participants have been described previously where the cohort was shown to represent a diverse population [24]. This study was approved by the Norfolk Local Research Ethics Committee (05/Q0101/191) and East Norfolk and Waveney NHS Research Governance Committee (2005EC07L). Participants gave signed informed consent at both baseline and then subsequently at the 3HC to cover new measures that were not present in previous health examinations.

**Table 1:** **Characteristics of the men and women in the EPIC-Norfolk 3 cohort**

Characteristic	Men (N = 3861)	Women (N = 4762)
**Mean (SD)**		
**Age**	69.4 ( 8.1)	68.1 (8.0)
**Mental activity score** (from Questionnaire)	21.1 (4.7)	23.2 (4.7)
**Frequency% (N)**		
**Education level**		
Left school with no formal qualification	22.2 (857)	29.7 (1412)
Left school with at least O level or equivalent	77.8 (3003)	70.3 (3349)
**Social class**		
I-III Non-manual	64.5 (2472)	67.2 (3165)
III Manual-V	35.5 (1362)	32.8 (1544)
**Smoking status**		
Current	4.2 (159)	4.5 (213)
Former	57.8 (2198)	36.4 (1711)
Never	38.0 (1446)	59.1 (2774)
**Physical activity**		
Inactive	37.4 (1422)	37.2 (1748)
Moderately-inactive	25.1 (954)	32.2 (1513)
Moderately-active	18.7 (713)	16.9 (796)
Active	18.8 (714)	13.6 (641)
**Employment and social activity**		
Retired from main occupation	75.9 (2855)	78.8 (3562)
Active in social groups	59.5 (2264)	68.5 (3219)

SD = Standard Deviation, Frequency N = Number.

The Cognition battery used in EPIC-Norfolk 3 (EPIC-COG) comprised of a number of validated tests that have all been described previously. These included a shortened version of the Extended Mental State Exam [25] (SF-EMSE), letter cancellation task [26] as used in the Medical Research Council Cognitive Function and Ageing Study (MRC CFAS) [27], Hopkins Verbal Learning Test (HVLT) [28], Cambridge Neuropsychological Test Automated Battery Paired Associates Learning Test (CANTAB-PAL) [29–31], Visual Sensitivity Test (VST) to assess visual impairment deficits contributing to cognitive impairment [32] Short National Adult Reading Test (Short-NART) [33] and a test for prospective memory (also as described in MRC CFAS) [34]. These tests are briefly described here with further information given in the Additional file 1. The tests were selected with the intention to cover an array of cognitive domains and a range of difficulty. Modifications were made to shorten some of the tests to allow for use in an epidemiological setting. The battery was part of a broader health examination that lasted approximately 2–3 hours depending on the participant.

## Cognitive tests

### Short Form Extended Mental State Exam (SF-EMSE)

The Extended Mental State Exam (EMSE) [25] extends the widely used Mini Mental State Exam (MMSE) [35], a test known for its limitations [36, 37], in particular in higher functioning individuals [25]. The original EMSE consists of 47 items in total including items from the Cambridge Mental Disorders of the Elderly Examination (CAMDEX) interview schedule [38] as well as items recommended in the report from the MRC Alzheimer’s Disease Workshop in 1987 [39]. Here, we used a modified shorter version consisting of 26 selected items assessing functioning at the higher end of the ability range.

### Short Form Mini Mental State Exam (SF-MMSE)

The SF-MMSE predicts the full-scale MMSE score by assuming an almost perfect performance on the excluded items in a highly functioning population [40]. The ‘full derived’ MMSE score (SF-MMSE Score +14) was used in the analysis here to allow the comparison of the other components of the battery using the SF-MMSE scores as a validated and recognised standard.

### Attention and visual search (Letter Cancellation)

This task involved a visual search of a set of random letters with the aim of crossing out as many of the 72 possible target letters (P and W) within one minute. The outcome measure was ‘Accuracy Score’ (number of correctly identified target letters minus all potential target letters missed).

### Hopkins Verbal Learning Test (HVLT)

Participants were asked to memorise words presented on a computer screen. At the end of the presentation participants were asked to recall the words. The list was shown a further two times. Correctly recalled words were recorded and the combined score of all three trials (Total HVLT Score) was used as the outcome measure.

### Cambridge Neuropsychological Test Automated Battery Paired Associates Learning Test (CANTAB-PAL)

The Paired Associates Learning Test (CANTAB-PAL), tests episodic memory and new learning and has shown to be a sensitive tool as a determinant of memory deficit in the early stages of dementia [30, 31, 41, 42]. Six white boxes (and then eight at the final stage) were presented on a touch screen, opening sequentially to display 1,2,3,6 and then 8 abstract visual patterns. Immediately after the final test pattern was displayed, one of the patterns was displayed in the middle of the screen and the participant was required to touch the box where that pattern was located on the screen. The task consisted of eight stages and up to ten presentations after which the task terminated. The outcome measure used here was the ‘first trial memory score’ (FTMS), the number of patterns correctly associated to their locations in the first attempt summed across the stages completed.

### Visual sensitivity test (reaction time)

This test consisted of two parts: Firstly, a triangle appeared randomly on the screen and the participant was required to press the space bar on the computer as soon as the triangle was seen. In the second part, a triangle formed from a screen full of moving dots and again the participant had to press the space bar as soon as the triangle was seen. The outcome measure of reaction time (in milliseconds) was recorded and stored automatically under the participant’s unique study number.

### The National Adult Reading Test (NART)

The National Adult Reading Test (NART) [43] shown to correlate with pre-morbid intelligence and general cognitive ability [44, 45], is accepted in both clinical and research settings. It is known to have some limitations, particularly in the less educated [46]. The participant was required to correctly pronounce the list of NART words presented on a computer screen. In this study, the short NART protocol [33, 47] was used. The outcome measure was ‘NART Error Score’, where a higher score indicates lower performance.

### Prospective memory

This is a test for the memory for future intentions, previously suggested to be sensitive to early stages of cognitive decline [34, 48]. Participants were asked to remember to carry out an explicit instruction at a specified point later in the appointment. Participants were defined as being ‘successful’ if they carried out at least one correct action without having to be prompted.

### Covariates

Data on covariates were obtained from a self-administered questionnaire. Smoking status, mental activities, employment status and hobbies were obtained from responses from the health questionnaire completed near the time of EPIC-Norfolk 3. The mental activity score was calculated by assigning 1 point for an individual who reported doing a particular activity once a year or less up to 5 points if they did the activity every day. In total there were seven activities (listening to the radio, reading the newspaper, reading magazines, reading books, playing games such as cards or chess, crosswords and puzzles). The minimum score possible was 7 and the maximum was 35. Those with missing data were excluded from the analysis.

Education level was obtained from baseline questionnaire and was coded into two categories: The first consisted of those leaving school with no formal qualification (less than O level or equivalent). The second category consisted of those leaving school with at least some qualification. The qualification group combined those attaining O-level or equivalent (completing school to the age of 15), A-level or equivalent (completing schooling to the age of 17 years) and those obtaining a degree or equivalent.

### Missing data and extreme outliers

If a test was abandoned or the participant refused to continue, the participant was scored on what had been completed and the data included in the analysis. Reasons for refusal were recorded to differentiate those participants who refused or failed to complete as a result of a technical fault or ran out of time, from those who refused because they expressed anxiety or difficulty with the task. Those who refused prior to starting a test or those who said no to a test component were assigned as missing data. Any participant identification number that could not be accurately assigned to a known individual was also removed from the final analysis as were any implausible values described below.

### Statistics

The outcome measures of six of the test components were continuous. The prospective memory variable measure was dichotomised into ‘successful’ and ‘unsuccessful’. The descriptive data (using the original untransformed scores of the continuous variables) are presented as medians and inter-quartile ranges as the cognitive scores in EPIC-COG were not normally distributed (although means and standard deviations are also presented in the Additional files 2 and 3: Table S1-S2). For the SF-EMSE Items, letter cancellation (Accuracy Score), HVLT, and CANTAB-PAL (FTMS), a higher score indicated better performance. The outcome measure for VST (reaction time) and for the NART (Short NART-Error Score), a higher score indicated poorer performance. Cross sectional data are presented by age and sex and by age, sex and MMSE Category. To give further details of the data, a graphical representation of the scores using a range of percentiles (1st, 10th, 25th, 50th, 75th, 90th and 99th) by age group and sex are also provided. Statistical analysis was performed using SPSS version 21.0 (IBM Corp., Armonk, NY, USA).

## Results

Of the 8623 participants attending EPIC-Norfolk 3, 45% (n = 3861) were men and 55% (n = 4762) were women. Cognitive data were collected on 8585 individuals, with over 90% completing or attempting six or all seven of the tests in the battery. The CANTAB-PAL and VST had slightly lower completion rate (at 86.5% and 83% respectively), partly due to a technical computer failure, resulting in loss of data on 150 participants. Table 2 summarises the cognitive domains covered by each of the tests used in this study and the number of participants completing each test component.

**Table 2:** **Summary of test components of the EPIC-COG battery and the number of participants who successfully attempting all or part of each component**

Cognitive test	Cognitive domain	Outcome measure* (Maximum possible test score)	Number of participants attempted/completed test component
**Short form-extended mental state exam (SF-EMSE)**	Global measure of cognition from MMSE to asses domains for retrospective memory (immediate and delayed), attention and calculation, registration, verbal registration, language (object naming/sentence), visual and constructional skills, praxis. Added items for Memory (extension on retrospective memory), praxis, verbal fluency (animal naming), language (writing to dictation) and abstract thinking.	SF-EMSE Score (37)	98% (8483)
**Letter cancellation task**	Executive function - (covering visual search, attention, mental and processing speed)	Accurately identified target letters in one minute (72)	97.5% (8410)
**Hopkins verbal learning test (HVLT)**	Recognition/learning and episodic memory	Total HVLT Score - Total of correctly identified target words over 3 trials (36)	93.7% (8081)
**CANTAB®-**paired associate learning (CANTAB-PAL)	Episodic memory and new learning/Visuospatial	First Trial Memory Score (26)	86.5% (7281)
**Visual sensitivity test (VST)**	Visuospatial (magnocellular pathway)	Reaction Time in milliseconds	83% (7144)
**National adult reading test (NART)**	Proxy Measure of IQ - Pre-morbid Intelligence	Short NART Error Score - Using the short-NART algorithm (50)	94.1% (8112)
**Time and event based task**	Prospective memory	Success vs Failure	97% (8403)

*Frequencies for only one outcome measure given in this summary table.

**Figure 1:**
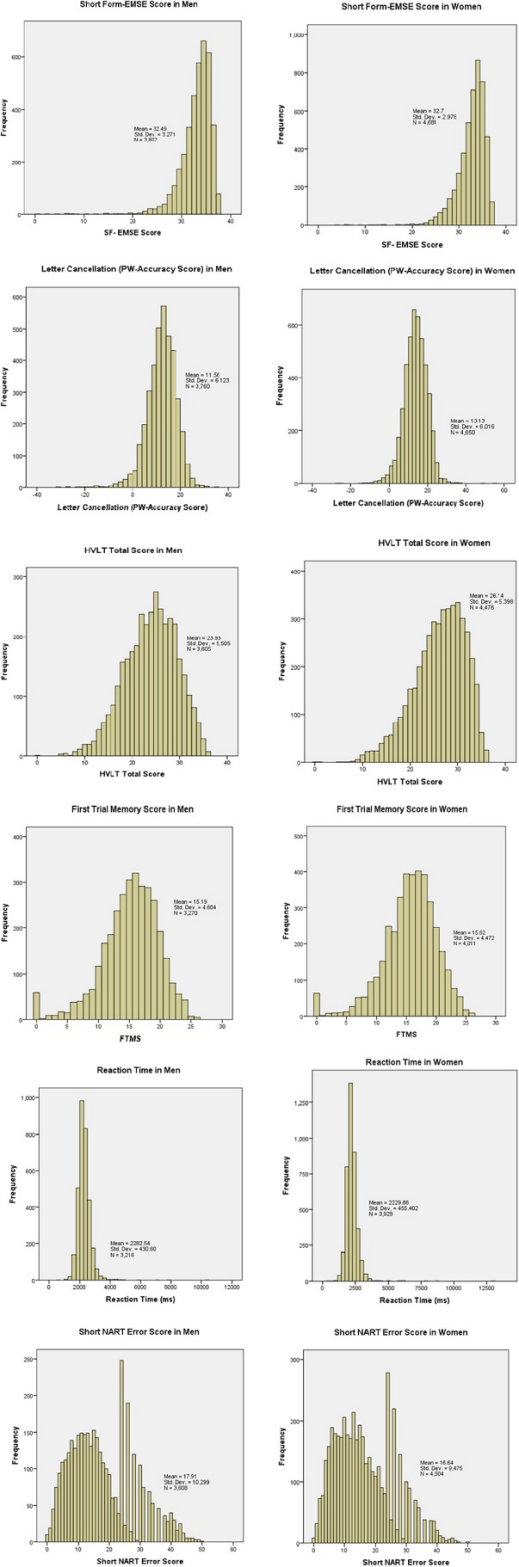
**Distribution of scores (of continuous variables) in men and women in EPIC-Norfolk 3.**

Men and women were equally likely to complete all seven components, with 6011 (69.7%) participants attempting all seven test components of EPIC-COG and only 850 participants (less than 10%) of those taking part attempting or completing five components or fewer. Those completing all seven components were slightly younger (mean age 68.8 compared to 70.9 years in men and 67.4 compared to 69.8 years in women), and were more likely to be either in paid employment or actively taking part in regular social networks when compared those who did not complete all the tests. More men had left school with qualifications than women (77.8% compared to 70.3%), although more women participated in regular social activities (68.5% for women compared to 59.5% for men). Women also reported more mental stimulating activities in their leisure time (with mean mental activity score of 23.2 in women as compared to 21.1 for men).Distributions for cognitive function (by each test component and stratified by sex) are presented in Figure 1. The EMSE distribution had a negative skew but did not have the same strong ceiling effect as the SF-MMSE scores in this cohort (distribution not shown), with 2298 (27%) of participants scoring the maximum SF- MMSE score of 29 as compared to only 2.4% (n = 200) scoring the maximum EMSE score of 37. The data for letter cancellation (PW Accuracy) and HVLT Total Score were both approximately normal distributed, as was the distribution for FTMS (other than a peak at score 0, which suggests that a high proportion of participants were unable to achieve a correct response immediately). The data for reaction time of the VST were highly positively skewed (as a result of a few extreme, but genuine slow responders).

**Figure 2:**
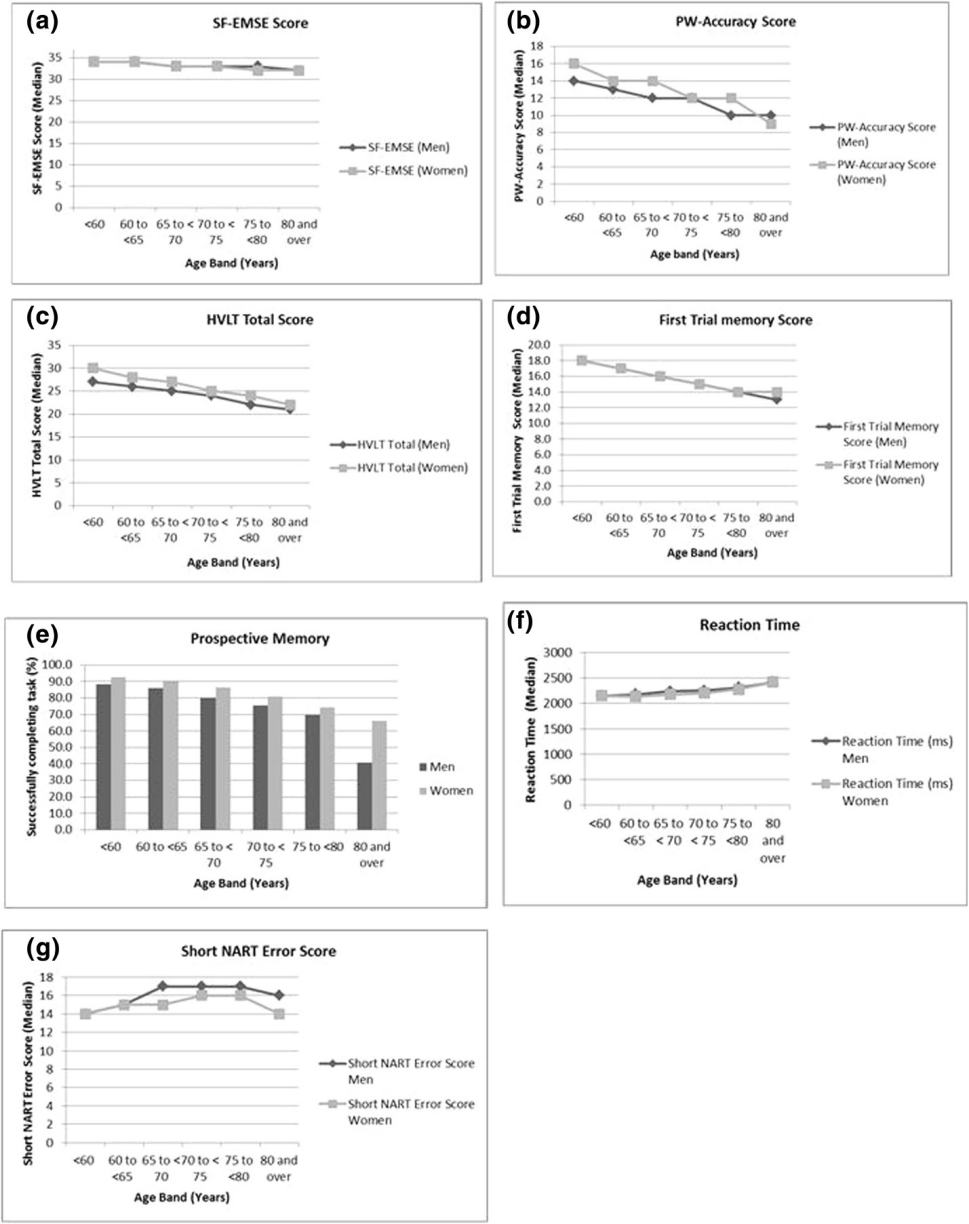
**Cognition performance with age in men and women. 2a-2d** are cognition test scores where higher scores indicating better performance and figures **2f** and **2g** are error scores where higher scores indicating poorer performance. Median values shown for the continuous variables and for prospective memory (figures **2e**) shown as percentage of the participants successfully completing task.

**Figure 3:**
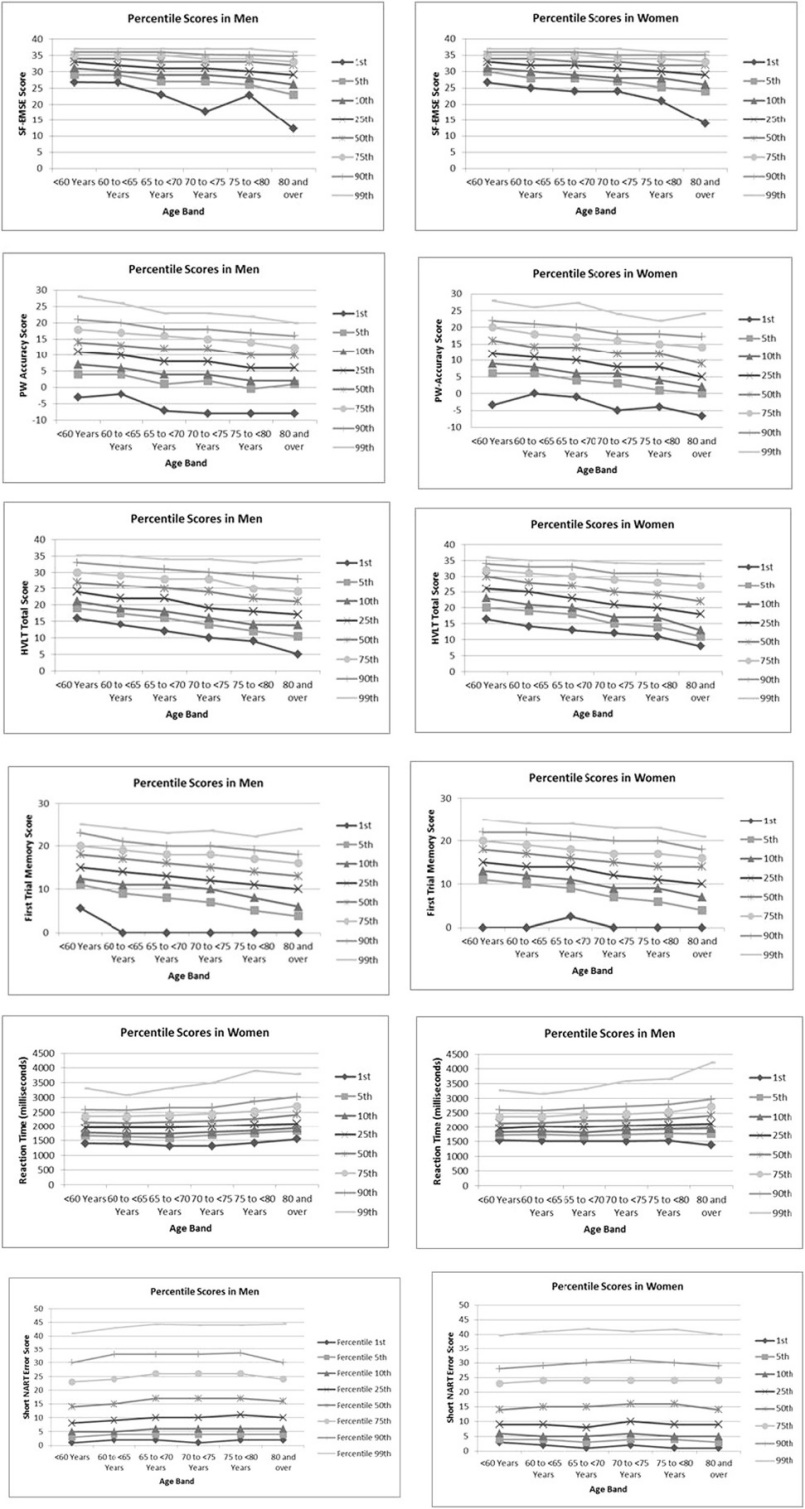
**Cross sectional percentile levels of scores by age group in EPIC-Norfolk men and women.** A graphical presentation of percentile scores plotted against age group (cross-sectional) showing performance from the highest to the lowest percentile scores in the six components of the EPIC-COG battery with continuous scores.

For the short NART, there was a peak at the error score of 24, followed by alternating peaks and troughs in data giving a ‘comb’ distribution. This pattern in the distribution is as a result of the short NART algorithm (as described in the Additional file 1). The peaks in the distribution can be attributed to those assigned an error score by the algorithm, artificially inflating the scores at these points. The greatest peak (and the starting point of this comb effect of the data) was seen to occur at the cut-off point of score of 20 (giving a full NART error of 24), which was the point where participants with this score or lower, did not continue with the second half of the test.The median scores of each of the test components were plotted with age in men and women as shown in Figure 2a-2g. The data presented here are cross-sectional values showing the association of scores with age group. The graphical presentations in Figure 2 indicate that median scores decline with age. The proportion of participants successfully completing the prospective memory task lowers with increased age group. In the case of the VST (Figure 2f), the median reaction time increases with age group. The NART error score showed an increase with age initially, remains steady then a slight reduction in the oldest group (Figure 2g). In almost all tests, women generally performed better than men.The data were further characterised by calculating percentile scores plotted by age group (Figure 3). In this figure, lower scores are seen across increasing age group. Higher percentiles of cognitive scores remain reasonably stable across age groups, but the spread and variation in scores becoming greater across each age group, with the lowest percentile having markedly lower performance. The variation in scores was least for the SF-EMSE compared to the other tests in the battery. For reaction time and short NART error score, the 99th percentile indicated the poorest performers. The short NART error score exhibited widest variation across age and even some improvement in scores in the oldest age groups.

### Comparability of test components with the widely accepted MMSE

The measures from each test component were compared to the MMSE, which is widely used in both research and clinical settings. The accepted cut off score of less than or equal to 23 as indicating presence of cognitive impairment has evolved from research findings [37] although higher cut-offs have been used [49, 50]. In this high functioning cohort, there were very few individuals with a score of ≤23; therefore the cut off used here for poorest performance was based on the 10th percentile score of 24. We used our modified form of the MMSE (i.e. SF-MMSE) and created three categories based on the distribution of SF-MMSE scores in the study population. The first category was defined as ≤ 24), the third category was defined at the highest SF-MMSE scores (28 and 29) and the middle category was created using the remaining scores of 25–27.

Tables 3 and 4 shows the distribution of all the tests in EPIC-COG across the three SF-MMSE categories in men and women respectively. The general trends for all the test components were similar with the scores of the continuous test variables improving across SF-MMSE categories. For the prospective memory test, the proportion of participants successfully completing the task also increased across the three SF-MMSE categories. Again, scores in women were slightly higher than men. There was still a range of performance seen across all three SF-MMSE categories.

**Table 3:** **Distribution of cognition test component scores by MMSE cut off- score in men**

Test	MMSE score ≤ 24				MSSE score 25-27				MMSE score 28-29			
	Frequency (n)	Mean (SD)	Median (IQR)	Range	Frequency (n)	Mean (SD)	Median (IQR)	Range	Frequency (n)	Median (SD)	Median (IQR)	Range
SF-ESME	240	25.2 (5.4)	27 (24, 28)	0, 32	1489	31.3 (2.2)	32 (30, 32)	17, 35	2073	34.2 (1.6)	34 (33, 35)	26, 37
P-W accuracy score	222	6.5 (7.9)	8 (3, 12)	−28, 20	1474	10.5 (5.9)	11 (7, 14)	−23, 31	2064	12.8 (5.6)	13 (10, 17)	−31, 34
HVLT	209	19.1 (5.9)	19 (15, 23)	0, 33	1391	22.4 (5.3)	23 (19, 26)	5, 35	1980	25.5 (4.9)	26 (22, 29)	7, 36
FTMS	195	11.9 (4.9)	13 (9, 15)	0, 23	1258	14.3 (4.6)	15 (12, 18)	0, 26	1794	16.2 (4.2)	17 (14, 19)	0, 26
VST (ms)	190	237 (733)	2263 (2063, 2509)	1239, 10489	1222	2310 (427)	2260 (2056, 2506)	567, 7148	1777	2253 (382)	2214 (2025, 2442)	1232, 7838
NART error score	200	25. 2 (11. 1)	26 (17, 33)	2, 50	1394	20.1 (10.4)	19 (12, 26)	0, 49	1985	15.6 (9.4)	14 (8, 22)	0, 49
Prospective memory (% at least one action correct)	217	55%			1470	73%			2063	84%

**Table 4:** **Distribution of cognition test component scores by MMSE cut off- score in women**

Test	MMSE score ≤ 24				MSSE score 25-27				MMSE score 28-29			
	Frequency (n)	Mean (SD)	Median (IQR)	Range	Frequency (n)	Mean (SD)	Median (IQR)	Range	Frequency (n)	Median (SD)	Median (IQR)	Range
SF-ESME	390	26. 8 (3.8)	28 (26. 29)	4, 32	1718	31.5 (2.0)	32 (30, 33)	20, 35	2573	34.4 (1.5)	35 (34, 35)	24, 37
P-W accuracy score	375	9.3 (6.4)	10 (613)	−16, 35	1710	12.3 (5.8)	12 (9, 16)	−28, 32	2565	14.3 (5.8)	14 (11, 18)	−26, 54
HVLT	352	21.1 (6.0)	22 (17, 25)	5, 36	1612	24.6 (5.3)	25 (21, 29)	0, 36	2467	27.9 (4.5)	29 (25, 31)	10, 36
FTMS	325	13.2 (4.5)	13 (11, 17)	0, 24	1433	14.8 (4.6)	15 (12, 18)	0, 26	2212	16. 6 (4.1)	17 (14, 19)	0, 26
VST (ms)	302	2393 (825)	2294 (2076, 2538)	459, 12869	1408	2259 (447)	2216 (2004, 2466)	1054, 7973	2167	2184 (374)	2156 (1965, 2374)	710, 8695
NART error score	358	24. 3 (10.4)	24 (16, 31)	2, 50	1616	18.6 (9.4)	18 (11, 26)	0, 47	2487	14.3 (8.4)	13 (8, 20)	0, 45
Prospective memory (% at least one action correct)	379	64%			1707	79%			2567	90%		

The top performing 2298 participants who performed perfectly on the SF-MMSE (achieving a maximum possible score of 29), also included participants who were amongst the poor performers (with scores in the bottom 10th percentile) for the other components. These findings were still valid for the top 25% and for the top 10% of the SF-EMSE performers (Table 5), although the numbers were increasingly lower than those seen with the top SF-MMSE scores.

**Table 5:** **Distribution of scores in participants with near perfect SF-MMSE and SF-EMSE scores**

	(A) Top MMSE score of 29				(B) Top 25% EMSE score (≥35)				(C) Top 10% EMSE score (≥36)			
	Men		Women			Men	Women			Men	Women	
	N*	Frequency% (N)	N*	Frequency% (N)	N*	Frequency% (N)	N*	Frequency% (N)	N*	Frequency% (N)	N*	Frequency% (N)
P-W accuracy score (10th percentile < = 5)	1006	8.2 (82)	1286	5.3 (68)	1034	5.5 (57)	1336	4.2 (56)	418	2.9 (12)	569	3.1 (18)
HVLT (10th percentile < =18)	962	6.7 (64)	1238	2.4 (30)	998	4.6 (46)	1285	1.1 (14)	404	1.5 (6)	568	0.5 (3)
FTMS (10th percentile < = 10)	870	6.2 (54)	1122	4.2 (47)	895	5.1 (46)	1149	3.6 (41)	360	5.3 (19)	514	2.5 (13)
VST (10th percentile > =2702 ms)	874	7.8 (68)	1100	5.4 (59)	904	7.1 (64)	1131	4.6 (52)	366	5.2 (19)	509	4.5 (23)
Short NART error score (10th percentile > =31)	964	5.9 (57)	1252	2.6 (33)	1006	4.0 (40)	1308	1.7 (22)	409	1.7 (7)	577	1.2 (7)

Participants achieving (A) Perfect MMSE Score of 29 (B) Top 25% SF-EMSE score and (C) Top 10% SF-EMSE score and scoring in the bottom 10th percentile of the other cognition tests.

N*: Total Number of Participants with both test scores available.

N: Number of participants in the bottom 10th percentile.

**Figure 4:**
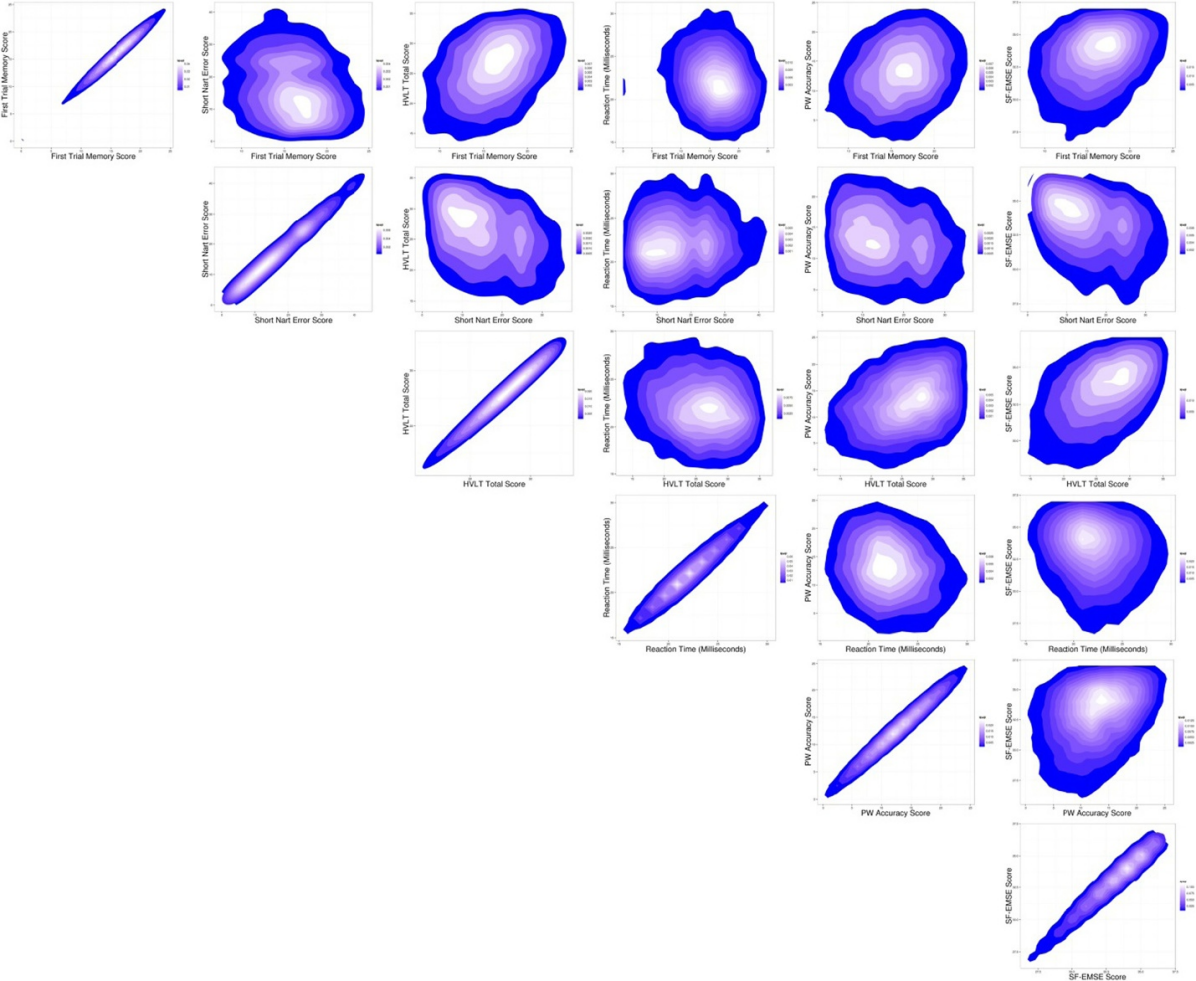
**Contour plots to show relationship of data from EPIC-COG battery.** The contours represent the strength of the relationship between the scores of the test components. The first plot (on the left) in each row shows the outcome measure variable of each individual test plotted against itself depicting the perfect positively linear association.

Spearman’s rank correlation coefficients were calculated (Table 6) to investigate the strength of relationship between each of the tests used in EPIC-COG. The correlations were moderate to weak for most tests with HVLT having stronger associations with the other tests, such as with SF-EMSE (r = 0.49) followed by FTMS (r = 0.38) and short NART (r = −0.38). This inverse association was as a result of the NART Error scale, with larger number representing poor performance.To illustrate the relationship of the tests further, the distribution of the scores were plotted as contour plots (Figure 4). The contours represent the strength of the relationship between the scores of the test components. The first plot in each row shows the outcome measure variable of each individual test plotted against itself depicting the perfect positively linear association. The peak (white area) representing the region with greatest density of scores, centres at different points for each test pair combination. There seemed to be some undefined spread for each test, however there was a systematic pattern seen in all the plots, with some of the test pairs showing a better relationship than others. The general direction of the plot and the peak of overlap of scores seem to appear in areas where one would expect, however with most of the plots what is seen at best is a moderate relationship between these tests.

**Table 6:** **Spearman’s correlation coefficient between the test components of the EPIC-COG battery**

	SF-EMSE	PW accuracy	HVLT	FTMS	Prospective memory	VST
PW-Accuracy	0.33					
HVLT	0.49	0.33				
FTMS	0.34	0.26	0.38			
Prospective memory	0.26	0.19	0.23	0.21		
VST	−0.16	−0.17	−0.17	−0.17	−0.09	
NART	−0.38	−0.21	−0.34	−0.21	−0.13	0.06

## Discussion

In this cross-sectional study, we report on cognitive function profiles across a range of domains using previously validated instruments in a general population of men and women from mid to later life (age 48–92 years). We focused mainly on age related differences in cognition measures, and found that, despite the EPIC-COG being a relatively long battery, it was well tolerated by this general study population.

Individuals can have impairment in one cognitive domain but perform well in another or a number of cognitive deficits can occur concurrently. There is increasing evidence of substantial variability in cognitive abilities within individuals. Hilborn et al., (2009) termed this variability in performance across different tasks within an individual as ‘dispersion’. It is important to gain a better understanding of the dispersion displayed by healthy individuals in order to allow accurate clinical judgment on unhealthy or abnormal dispersion [51].

Previous studies have shown that a variety of cognitive deficits are associated with preclinical stages of different types of dementias and that decline can occur in a number of cognitive domains, even before any of the clinical criteria of early stages of cognitive decline are met [52–54]. Our findings are consistent with previous studies that have shown variability and dispersion across different cognitive domains [51, 55] in older people. Further investigation is necessary to confirm whether the differences observed across the domains provide any meaningful indicators of cognitive performance over time.

The frequency distribution and data from the pilot (where the full NART was used for 300 participants) indicate that the short NART equation does not hold so well in this heterogeneous ageing population as it did in the homogenous sample of elderly women living in a rural setting that it was initially tested on. The aim of the short- NART was to lessen participant load and anxiety, however in an assessment such as the one in this study; participants were as likely to be anxious for any of the other components. The pilot data show that participants who obtained a score of 20 (n = 82) for the first half of NART obtained a mean score of 7.9 (SD = 3.7) for the second half, which is better than what the algorithm predicts. Therefore, we would recommend that despite the appeal of the short NART, this protocol is not appropriate for a higher functioning population where few individuals have poor reading skills. If there are concerns about causing distress, then we would suggest lowering the cut-off to at least 17 rather than having the cut-off at 20.

Prevalence of severe cognitive impairment is relatively low in our cohort but as with previous studies [56, 57], we also found age to be inversely associated with the complete range of cognitive function being tested with the exception of NART, where there did not seem to be any strong association between the short NART score and age. This confirms previous findings that NART is a good measure of pre-morbidity [44, 45] and that age has little or no effect on NART performance in the absence of early dementia [58].

We observed some differences in abilities across gender and age categories. These may have arisen because of age and sex differences in education status. Although we have not investigated education in detail here, there is good heterogeneity of individuals leaving school with and without qualifications in all age groups analysed. We also observed a trend not noted in other studies, of women performing better than men in all the test components across all age groups, even though more men than women reported leaving school with some qualification suggesting that educational status would not explain the sex differences seen in this cohort. However, women reported to be more socially active and to be doing more mentally stimulating activities in their spare time, which have been linked to better cognitive performance at older age. The effects of education, social networks and mental activity will be the focus of further research.Although the test scores generally decline across age groups with the widest variation seen in the oldest age group, there is still a range of capability from poor to high performance in each age band, with some participants from the oldest age group outperforming their younger counterparts. Percentile scores from our cross-sectional data (Figure 3) show that the greatest decline in all test components is seen in poor performers across all the age groups. This graphical presentation can be used to compare scores or estimate age and sex adjusted scores across the different domains investigated here. Individuals scoring below the 25th percentile could be considered as cognitively impaired for that domain and require further investigation.

The MMSE’s lack of ability to assess individual domains and its poor sensitivity to mild cognitive impairments are frequently cited limitations [59, 60] This is because most healthy individuals can successfully answer most of the test items. Even though more demanding tests are available [25, 36, 60, 61], the MMSE still remains the most widely used and cited test of global cognition. To allow comparability with other studies, we included the SF-MMSE to provide a baseline for future studies and as a means of comparing the psychometric qualities and utility of the other tests used in the battery. The MMSE, being a global measure of cognition contains items that test the same domains of memory function as the other components of EPIC-COG. As expected, a positive trend was seen in all the tests with increased SF-MMSE score category, however, a range of scores were seen for other tests in each of the SF-MMSE categories.

On further investigation of participants obtaining the maximum score of 29 on the SF-MMSE, we found in the range of 6-8% of men and 2-5% of women also scored in the bottom 10th percentile of the other tests (Table 5). On examining those individuals who obtained the top 25% and further in the top 10% SF-EMSE scores, there were still participants in poorest performers (10th percentile) of the other tests, although the figures were reduced. Those scoring in the bottom 10th percentile tended to be older than those with scores above the 10th percentile for all the tests apart from the Short NART The number of participants scoring the maximum possible on the SF-EMSE was small (n = 200). None of these individuals scored in the bottom 10th percentile of the other tests other than one person who scored in the lowest 10th percentile for the NART. We show that obtaining a perfect score on the MMSE does not indicate absence of impairment and this confirms previous findings for the need to supplement the MMSE in cognitive testing [37], particularly in a normal to high functioning population.

The limited reliability and validity of the MMSE in a general population has been attributed to the restricted range of MMSE. The EMSE has been shown to be sensitive across a range of performance, to avoid the ceiling effect and that (even in its short form as it has been used here), the EMSE provides extended coverage of cognitive domains (extending on attention, memory, processing and executive function). This report confirms previous findings that the EMSE has advantages over the MMSE particularly for testing individuals at the high end of the performance range [25].

Spearman’s rank correlation (Table 6) show correlations are moderate to weak for most tests with HVLT having the strongest associations with other test components especially with FTMS and NART. This is not surprising, as HVLT, FTMS and NART assess similar cognitive sub domains of memory and language, however the moderate degree of correlation is somewhat counterintuitive as we would expect this to be higher. The contour plots (Figure 4) depict the spread of scores (and area of overlap) indicating that with some association, there is also some non-systematic scatter of scores suggesting that these tests may be assessing different aspects of cognitive function.

We have also addressed some practical and methodological issues with regards to minimising variability and subjectivity that can be introduced at any part of administration, scoring or cleaning of the data. We have described methods of standardisation in an epidemiological setting to ensure accuracy and consistency. Having these methods standardised and documented is also extremely important to allow comparability and potential harmonisation of data with other studies. The other advantage of this study over previous studies is that it has been conducted in a large well characterised cohort of men and women with good representation from a very wide age range, which has been a limiting factor in some previous studies [27, 62–64].

### Limitations to the study

The main limitations of this study relate to all cohort studies, and that is of healthy volunteer bias and attrition as highlighted previously [24]. However, although there is likely to be some loss of the more cognitively impaired, the oldest and frailest participants, there remains a wide range of performance and health across the whole age span of interest (from mid–life to over 90 years) represented in EPIC-Norfolk 3. The other limitation is that this is a cross-sectional study and so age differences and between persons effect can be observed but not within person differences or age related changes for which longitudinal data is required. Finally, and very importantly, we have not adjusted for the potential effects of education which is a known strong predictor of cognitive function [65–67] and on the rates of decline [68]. However, the purpose of this paper was not to look at lifestyle factors in any detail, but to present the descriptive data on this cohort. The effects of education and other factors on cognitive performance across different domains will be examined in future analysis.

## Conclusion

Everyday activities in the real world are complex, requiring independence, planning, organisation, sequencing and judgement and have been shown to be a significant predictor of functional status [69]. Therefore, assessing cognitive function in a range of domains such as executive functions, planning, flexibility, abstract thinking, semantic memory as well as episodic memory is vital. Also of considerable importance is to accurately identify early decline in individuals or those with MCI who are known to be at increased risk of dementia, particularly Alzheimer’s disease (AD) compared to older people without any obvious cognitive impairment.

Here we have used a comprehensive battery of accurate and well tolerated tests to provide evidence of cognitive function in a number of cognitive domains that have previously been reported to be involved in much earlier stages of decline. We have described how, even though there is reduction in performance across age, there is also a great deal of heterogeneity in older individuals. Further work is needed to understand why cognitive abilities vary so greatly across individuals and cognitive domains and to investigate the more subtle changes in cognition. We have also demonstrated that the EMSE even in its short form provides a better description of the cognitive abilities in a general functioning population and that the short NART protocol is not suitable for a heterogeneous higher functioning population. Finally, careful consideration should be given to the purpose for using a particular test (including whether the aim is to obtain global or domain specific measure, time availability and target population) when selecting an assessment tool for cognitive function.

There is epidemiological evidence of associations between lifestyle factors (such as diet, smoking and exercise) and risk of dementia [70]. The EPIC-Norfolk study has over twenty years of lifestyle, biological and genetic information, collected from mid to late in life. This study is well placed not only to identify factors associated with decline but also factors associated with maintaining abilities in older age. With the data already collected and further follow up data, we can investigate patterns of behaviours over time and predict how those behaviours affect cognitive function.

## Electronic supplementary material

Additional file 1: **Supplementary information.** Cognitive function in a general population of men and women: A cross sectional study in the European Investigation of Cancer and Nutrition–Norfolk cohort (EPIC-Norfolk). (DOCX 38 KB)

Additional file 2: Table S1: Cognitive scores stratified by age in men in EPIC-Norfolk 3. (XLSX 23 KB)

Additional file 3: Table S2: Cognitive scores stratified by age in women in EPIC-Norfolk 3. (XLSX 25 KB)
