# Supplementary material for: Cognitive function in a general population of men and women: a cross sectional study in the European Investigation of Cancer–Norfolk cohort (EPIC-Norfolk)
Source: BMC Geriatr. 2014 Dec 19;14:142. doi: 10.1186/1471-2318-14-142 (PMC4349767; doi:10.1186/1471-2318-14-142)
Supplement: Supplementary file 1 — Additional file 1: Supplementary information. Cognitive function in a general population of men and women: A cross sectional study in the European Investigation of Cancer and Nutrition–Norfolk cohort (EPIC-Norfolk). (DOCX 38 KB) [file 12877_2014_1084_MOESM1_ESM.docx]

SUPPLEMENTARY INFORMATION

**Title:** Cognitive function in a general population of men and women: A cross sectional study in the European Investigation of Cancer and Nutrition –Norfolk cohort (EPIC-Norfolk).

**Shabina A Hayat, Robert Luben,Stephanie Moore, Amit Bhaniani, Serena Anuj and Nichola Dalzell**

**Protocol**

**Recruitment and training of staff**

Cognitive function was assessed as part of the EPIC-Norfolk Cohort Study Third Health Examination (EPIC-Norfolk 3) [1]. The health examination lasted approximately 2.5- 3 hours of which the cognitive battery could take up to 40-50 minutes depending on the participant. Nurses employed for this study were professionally qualified with experience of performing phlebotomy. Intensive three month training was provided initially with regular refresher courses throughout the year and quality assurance at regular intervals to assess for internal consistency and inter-rater reliability. Nurses were trained to follow the detailed standard operating procedures (SOPs) and where necessary adhere to script, with standard verbal responses and prompts to ensure consistency and accuracy. The protocol also included guidance on how to encourage participants without giving feedback. Detailed guidelines for scoring (which can be found in the technical report available from the EPIC-Norfolk website at <http://www.srl.cam.ac.uk/epic/TechnicalReports>) were also provided. The cognition battery was conducted approximately halfway through the appointment. This was so that sufficient time had elapsed for the nurse administering the test to establish a rapport with the participant, but not too long into the appointment where the participant may have become anxious or tired, which could then have had a possible effect on their performance.

**Data Entry and Data Checking**

Data was captured directly on an electronic case report form (eCRF) which was printed out and then used to record the paper and pencil items of EPIC-COG. The scores from the computerised tests were stored automatically under the participant’s unique EPIC Study identifier. There was a three staged process for checking the data. The first stage was carried out at the end of the appointment by the nurse carrying out the health examination; this was to ensure that most issues involving missing data were corrected immediately. The second stage was done by a different nurse (following a checklist) to investigate and correct any discrepancies in the CRF and where possible, make the relevant amendments on local machines (e.g. ID number typos etc.). All amendments were noted. To further verify the data entered, a third stage checking- was carried out on an ad-hoc basis by clinic manager or by a member of the research team based in Cambridge.

**Consistency in test administration**

As variability and subjectivity can be introduced at any part of administration, scoring or cleaning of the data, emphasis was given to standardising the protocol minimising differences in interpretation and subjectivity. The protocol included clear explicit guidelines with verbatim instructions where possible. This was particularly useful for tests that are widely used such as the NART [2,3] which is subject to inter-rater variation [4] and the MMSE [5] which have been reported to lack specificity [6,7]. The MMSE is also subject to variability, because there is no accepted documented method for administration and scoring. To ensure accuracy, consistency and to enhance nurse confidence, training from experts within the field was given for each component regularly throughout the study.

For completeness and accuracy of data, participants were kept motivated and encouraged to complete the battery, however, participants were free refuse any part of the heath check. Refusals and reasons for aborting were recorded (although complete data not available at the time of writing) so that comparisons between those with complete data and those with missing data could be made. Data was analysed as it was collected to record common responses given which were included in drop down menus as possible responses to reduce nurse interpretation. Also for completeness several checking measures were included at various stages for data entry and cleaning. Where there was a discrepancy in the scoring between two researchers, the data was analysed by a third person, and the final decision was made by consensus.

**Reporting on results**

Participants scoring low on any of the cognition tests were not referred for further assessment, nor were results reported to their GP. This was indicated on the participant information sheet (PIS) sent to the participant in the invitation pack. Clinically relevant results from other parts of the health examination such as for raised blood pressure, lipid profile and the eye examination were referred to GP Practices for further follow-up.

**Recruitment of Participants**

At baseline between 1993 and 1997 [8], participants on the registers of thirty-five general practices were invited to take part in EPIC-Norfolk, of which 30 445 consented and completed a health questionnaire and of those 25 639 attended a health examination. For EPIC-Norfolk 3, only participants consenting at baseline (those who had not requested to be removed from future approaches and were still alive at the time of approach) were invited. Practices were approached two at a time, based on geographical location and distance from the clinic in Norwich, i.e. one at close proximity (city practice) and one further afield (rural area). Funding constraints led to the exclusion of four practices from follow-up. As a result of not including these practices, 1517 eligible participants were not approached however this exclusion did not introduce bias into the study [1]. Record linkage to the NHS Exeter System ensured participant contact information was up to date at the time of approach.

**Consent**

All EPIC participants consented at baseline to participate in the long term study. Consent was taken again from participants taking part in EPIC-Norfolk 3 to cover new procedures including the cognitive tests that were not applied at previous health examinations and to update the consent to comply with current standards. In the invitation, participants were asked to read the Participant Information Sheet (PIS) relating to the study and were asked to complete a participation form indicating their willingness to take part and their preferred day and time for their appointment. They could also, at this point, refuse (and if they chose, give reasons for refusal). If the participant agreed to take part, they were asked to read the consent form carefully, but not complete it until they arrived at the clinic. Consent was then given in the presence of the nurse who explained the health examination to the participant, answered questions and ensured that the participant was completely informed of what they were being asked to do, before signing the consent form. It was also made clear to the participant that they could refuse any part of the health examination should they wish to do so.

**Cognitive Tests**

**Short Form Extended Mini Mental State Exam (SF-EMSE) and Short Form Mini Mental State Exam (SF-MMSE)**

The Extended Mental State Exam [9] (SF-EMSE), consisting of 47 items (giving a score of 60) extends on the Mini-Mental State Exam. A shortened version of the SF-EMSE (consisting of 26 items) giving a maximum score of 37 and containing items from the SF-MMSE was used in EPIC-Norfolk 3.

The SF-MMSE has been shown to predict the full-scale MMSE score by assuming an almost perfect performance on the excluded items in a highly functioning population [10]. It has been shown capture the range of scores from severely impaired to the high functioning. The SF-MMSE was demonstrated to be a useful instrument for screening large numbers of population studies, with the results comparable to studies where the full test version was administered. The ‘full derived’ MMSE score (SF-MMSE Score+14) has been used in the analysis here to allow the comparison of the other components of the battery using the SF-MMSE scores as a validated and recognised standard.

**Attention and Visual Search**

The letter cancellation task [11] assesses attention, mental processing and speed. The task involved a visual search of a set of random letters printed in a grid like format consisting of 26 rows and 30 columns with the aim of crossing out as many of the 72 possible target letters (P and W) within one minute (as used in MRC CFAS Study).[12] There are two outcome measures in this task, the first is for speed which is total numbers of letters searched in the allocated time and the second is the accuracy score (the outcome measure that was used here), which is number of correctly identified target letters minus all potential target letters missed up to the point scanned by participant.

**Hopkins Verbal Learning Test (HVLT)**

The HVLT [13] is a short reliable test of verbal recall and recognition. [14,15,16] The test consists of a 12-item word list, from three semantic categories, (‘precious stones’, ‘human shelter’ and ‘animals with four legs’). Participants were presented with the word list on a computer screen and asked to memorise the words. The computer program was timed with an interval of 1 second between each word presentation. At the end of the presentation the participant was asked to recall as many of the words as they could. The list was shown a further two times. Correctly recalled words were recorded. A score for each trial and the total recall score (maximum of 36) was noted. Any incorrect words mentioned were also documented, and although not used in here, are available for future analysis. Here, we used the total HVLT score as the outcome measure.

**Cambridge Neuropsychological Test Automated Battery Paired Associates Learning Test**

**(CANTAB-PAL)**

The Paired Associates Learning Test (CANTAB-PAL) shown to be a highly sensitive tool as a determinant of memory deficit in the very early stages of dementia,[17,18,19,20] was used here to assess episodic memory and new learning. Participants were presented with six white boxes (and then eight at the final stage) on a touch screen, opening sequentially to display 1,2,3,6 and then 8 abstract visual patterns. Patterns were displayed one by one in random box positions. Immediately after the final test pattern was displayed, one of the patterns was displayed in the middle of the screen and the participant was required to touch the box where that pattern was located on the screen. If the choice was correct, the procedure continued to the next set of patterns. If incorrect the boxes with the pattern(s) were presented again. The task consisted of eight stages and up to ten presentations. If all the patterns were not correctly placed by the tenth presentation, the task automatically terminated. There are a number of outcome measures in CANTAB-PAL, all which have to be analysed with reference to the PAL stages completed measure. Further explanation of this key indicator is given in the supplementary information. For the analysis here, the outcome measure used was the first trial memory score (FTMS), which is the number of patterns correctly associated to their locations in the first attempt summed across the stages completed.

**Visual Sensitivity Test**

The VST [21] assesses disturbance of the magnocellular pathway and consisted of two parts: In the first part, a triangle appears at random on the screen and the participant had to press the space bar on the computer as soon as the triangle was seen. In the second part, the screen is full of constantly moving dots, from which a triangle forms at a random point on the screen. The test required the participant to press the space bar when the triangle became apparent, with the triangle gradually becoming more obvious with time, and so increasing the probability of detection over time. The outcome measure of reaction time (in milliseconds) was recorded and stored automatically under the participant’s unique study number. The visual sensitivity test gave 70 measures per participant, with the final reading value (used in analysis) being the average of these measures. The data contained some extreme measures (method for identifying these values is given in the supplementary information).

**NART**

The National Adult Reading Test (NART) [22, 23] has been shown to correlate with pre-morbid intelligence and general cognitive ability. [24,25] The NART uses the assumption that the level of reading ability is closely related to general intellectual level and is a more objective measure of pre-morbid ability than using demographic variables such as years of education and socio-economic status. [25] The NART is a widely accepted and commonly used method in clinical and research settings, even though it is known to have some limitations, particularly in the less educated.[26] As the words in the NART do not follow the standard (British) grapheme-phoneme rules, the test requires the participant to recognise the word in its written form, obtaining this information from a lexical store, as decoding the word would result in an incorrect pronunciation. The NART thus gives an implicit measure of the individual’s knowledge of the English vocabulary. It aims obtain a measure of prior intelligence when assessing level of cognition for an individual both in absolute terms and relative to others within their age range.

The words were presented to the participant on a computer screen and required the participant to pronounce correctly a set of up to 50 irregular words of varying difficulty. The short NART protocol [27], from which the full NART score can be derived using an algorithm based on the performance on the first half of the test, was used (as explained in the supplementary Information). Only those individuals scoring between 21-25 on the first half of the test, proceeded to the full NART of 50 words. As the outcome here is an error score (Short NART Error Score), a higher score indicated lower performance. There have been some reservations regarding the practical utility of the short NART, but its accuracy has been shown to be almost equivalent to the full-length NART.[28]

**Prospective Memory**

This is memory for future intentions and has been previously suggested to be sensitive to early stages of cognitive decline.[29,30] In this task, participants were asked to remember to carry out an explicit instruction at a specified point later in the appointment. This was to seal and initial an envelope when it was handed back by the nurse later in the assessment. Responses were scored on whether both, one or none of the actions were completed when the envelope was handed to them.

For the purpose of the analysis here, participants were defined as being ‘successful’ if they carried out at least one correct action without having to be prompted by the nurse.

**Health and Lifestyle Questionnaire**

Health and lifestyle data were collected on the self-completed questionnaire, and included health variables (e.g. self-reported health, medications, physical performance, amount of physical activity, cognitive functioning, level of difficulties with activities of daily living and information regarding sexual function); psychological variables, (e.g. mental health, well-being, life satisfaction, mood); socio-economic variables (e.g. current work activity, reasons for retiring, financial security); and social activity (leisure activities).

The EPIC-COG battery was conducted approximately half way through the health examination. This was to allow enough time to build a rapport with the participant, yet not so long that there may be a possibility of the participant becoming too tired and losing concentration. This also gave the nurse time to evaluate the participant and ensure that they would understand and follow instructions given to them. Participants were asked to wear their glasses and hearing aid if they usually wore one. Any physical problems (such as visual impairment) or problems understanding instructions were noted by the nurse at the health examination. The time taken for the cognitive test varied depending on the cognitive ability of the participant, but took a minimum of 35 minutes to complete.

**References**

1. Hayat SA, Luben R, Keevil VL, et al. Cohort Profile**: A prospective cohort study of objective physical and cognitive capability and visual health in an ageing population of men and women in Norfolk (EPIC-Norfolk 3).** Int J Epidemiol. 2013
2. Nelson H. R. **National Adult Reading Test (NART): Test Manual.** NFER-Nelson, Windsor;1982
3. Nelson, H. E. & Willison, J.). **National Adult Reading Test Manual (2nd edn.)**. NFER-Nelson: Windsor 1991
4. Caroll RE. **The inter-rater reliability of the National Adult Reading Test (NART): A pilot study.** British Journal of Clinical Psychology 1987 **26** 229-20

**5** Folstein MF, Folstein SE, McHugh PR. **‘‘Mini-mental state.’’ A practical method for the cognitive state of patients for the clinician**. J Psychiatr Res 1975;**12**: 189–98.

6 Teng EL, Chui HC: **The Modified Mini-Mental State (3MS) examination** J Clin Psychiatry 1987 **48**: 314-318

7 Tombaugh TN, McIntyre NJ: **The mini-mental state examination: a comprehensive review.** J Am Geriatr Soc 1992, **40**:922-935.

8 Day N, Oakes S, Luben R et al. EPIC-Norfolk: **Study design and characteristics of the cohort. European Prospective Investigation of Cancer.** Br J Cancer 1999; **80** **(1):**95–103

9 Huppert FA, Cabelli ST, Matthews FE, and the MRC Cognitive Function and Ageing Study (MRC CFAS): **Brief cognitive assessment in a UK population sample – distributional properties and the relationship between the MMSE and an extended mental state examination.** BMC Geriatrics 2005, **5:7** doi:10.1186/1471-2318-5-7

10 Matthews FE, Stephan B.C.M, Khaw K T, Hayat S, Luben R, Bhaniani A, Moore S, Brayne C: **Full-scale scores of the Mini Mental State Examination can be generated from an abbreviated version**. J Clin Epidemiol 2011 **64** (9): 1005-13 Epub 2011 Mar 16

11 Richards M, Kuh D, Hardy R, Wadsworth M. **Lifetime cognitive function and timing of the natural menopause.** Neurology. 1999;**53** (2):308–14.

12 MRC CFAS: **Cognitive function and dementia in six areas of England and Wales: The distribution of MMSE and GMS organicity level in the MRC CFA Study.** The Medical Research Council Cognitive Function and Ageing Study (MRC CFAS). Psychol Med 1998, 28: 319-335

13 Brandt J. **The Hopkins Verbal Learning Test: Development of a new memory test with six equivalent forms**. The Clinical Neuropsychologist, 1991 **5**, 125-142

14 De Jager C, Hogervorst E, Combrinck M and Budge MM: **Sensitivity and Specificity of neuropsychological tests for mild cognitive impairment, vascular cognitive impairment and Alzheimer’s disease.** Psychological Medicine 2003 **33** 1039-1050

15 Frank RM, Byrne GJ: **The clinical utility of the Hopkins Verbal Learning Test as a screening test for mild dementia**. Int J Geriatr Psychiatry 2000 **15** 317-24

16 Hogervorst E., Combrinck M, Lapuerta P, Rue J, Swale K and Budge M. **The Hopkins Verbal Learning Test and Screening for Dementia.** Dementia and Geriatric Cognitive Disorders. 2001 **13** 213-220

17 Fowler KS, Saling MM, Conway EL, Semple JM, Louis WJ: **Paired Associates Performance in the early detection of DAT.** J Int Neuropsychol Soc 2002 **8** (1) 58-71

18 Fowler KS, Saling MM, Conway EL, Semple JM, Louis WJ: C**omputerized neuropsychological tests in the early detection of dementia.** J Int Neuropsychol Soc 2002 **3** 139-146

19 Swainson R, Hodges JR, Galton CJ, Semple J, Michael A, Dunn BD, Iddon JL, Robbins TW, Sahakian BJ: **Early detection and differential diagnosis of Alzheimer’s disease and depression with neuropsychological tasks**. Dement Geriatr Cogn Disord 2001: 12 265-280

20 Blackwell AD, Sahakian BJ, Vesey R, Semple J, Robbins TW, Hodges JR: **Detecting Dementia. Novel neuropsychological markers of pre-clinical Alzheimer’s Disease.** Dement Geriatr Cogn Disord 2004 **17** 42-48

21 Kirby, L., Bandelow, S, Hogervorst, E. **Visual impairment in Alzheimer’s disease Journal of Alzheimer’s disease** 2010 Feb 24 epub

22 Nelson H. R. **National Adult Reading Test (NART): Test Manual.** NFER-Nelson, Windsor;1982

23 Nelson, H. E. & Willison, J.). **National Adult Reading Test Manual (2nd edn.)**. NFER-Nelson: Windsor 1991

24 Crawford JR, Parker DM, Stewart LE, Besson JA and De Lacy G: **Prediction of WAIS IQ with the National Adult Reading Test.** British journal of Clinical Psychology 1989 **28** 267-273

25 Crawford JR, Deary IJ, Starr J and Whalley LJ: **The NART as an index of prior intellectual functioning: a retrospective validity study covering a 66-year interval.** Psychological Medicine 2001 **31** 451-458

26 Brayne C and Beardsall L: **Estimation of verbal intelligence in an elderly community: An epidemiological study using NART. British journal of Clinical Psychology,** 1990 **29;**21-223

27 Beardsall, L. & Brayne, C. **Estimation of verbal intelligence in an elderly community: A prediction analysis using a shortened NART.** British journal of Clinical Psychology, 1990 **29;** 83—90

28 Crawford JR, Parker DM, Allan KM, Jack AM and Morrison FM. **The Short NART: Cross-validation, relationship to IQ and some practical considerations** British journal of Clinical Psychology 1991, **30**, 223-229

29 Huppert FA, Beardsall L.. **Prospective memory impairment as an early indicator of dementia. Journal of Clinical and Experimental Neuropsychology**  1993 **15**: 805-821.

30 Huppert FA, Johnson AL, Nickson J, M**RC CFAS: High prevalence of Prospective Memory Impairment in the Elderly and in Early-stage Dementia: Findings from a Population-based Study.**  Applied Cognitive Psychology 2000, **14**: S63-S81
